# Supplementary material for: Genetically-stable engineered optogenetic gene switches modulate spatial cell morphogenesis in two- and three-dimensional tissue cultures
Source: Nat Commun. 2024 Dec 2;15:10470. doi: 10.1038/s41467-024-54350-7 (PMC11612184; doi:10.1038/s41467-024-54350-7)
Supplement: Supplementary file 1 — Supplementary Information [file 41467_2024_54350_MOESM1_ESM.pdf]

Genetically-stable engineered optogenetic gene switches modulate spatial cell morphogenesis in two- and three-dimensional tissue cultures

Supplementary Information

Authors

Hannes M. Beyer[1,#], Sant Kumar[2,#], Marius Nieke[1], Carroll M.C. Diehl[1], Kun Tang[1], Sara Shumka[1], Cha San Koh[1], Christian Fleck[3], Jamie Davies[4], Mustafa H. Khammash[2,@], Matias D. Zurbriggen[1,5,@]

Affiliations

[1]Institute of Synthetic Biology, Heinrich-Heine-University Düsseldorf, Universitätsstrasse 1, D-40225 Düsseldorf, Germany

[2]Department of Biosystems Science and Engineering (D-BSSE), ETH Zürich, Klingelbergstrasse 48, 4056 Basel, Switzerland

[3]Freiburg Center for Data Analysis and Modeling (FDM), University of Freiburg, Ernst-Zermelo-Straße 1, D-79104 Freiburg im Breisgau, Germany

[4]Deanery of Biomedical Sciences, University of Edinburgh, Edinburgh EH8 9XD, United Kingdom

[5]CEPLAS – Cluster of Excellence on Plant Sciences, Düsseldorf, Universitätsstrasse 1, D-40225 Düsseldorf, Germany

[#]These authors contributed equally to this article

[@]To whom correspondence should be addressed: mustafa.khammash@bsse.eth.ch and matias.zurbriggen@uni-duesseldorf.de

Supplementary Information content

**Figure S1**	Transposition-competent genetic constructs encoding red and blue light-responsive photoswitches for the optogenetic gene expression devices.
**Note to Figure S1**	Design of the tested optogenetic gene switches
**Figure S2**	Partial genomic integration of optogenetic gene switches, full dataset.
**Notes to Figure S2**	Notes on vector details.
**Figure S3**	Effects of blue and red light induction of necroptosis in bulk cultures.
**Figure S4**	Red light-induction of necroptosis in a selected clone of a CHO-K1 cell line using the RED_E gene switch in combination with the $MLKL_N$-encoding vector as in Figure 4A.
**Figure S5**	Spatial induction of necroptosis using a 3D-printed photomask.
**Figure S6**	Erythromycin supplementation protects the engineered CHO-K1 cells from inducing necroptosis upon blue light illumination via the DMD by displacing the E DNA-binding protein from the DNA operator sequence.
**Figure S7**	Unspecific induction of necroptosis due to extensive imaging.
**Figure S8**	Z-layers of the intensity projection images in Figure 5D.
**Figure S9**	Profile of randomly-selected clones of $HEK^{Cdh3\text{-}OptoWnt}$ cells harboring the $BLUE_{SINGLE}$ gene switch as in Figure 6A.
**Supplementary References**	

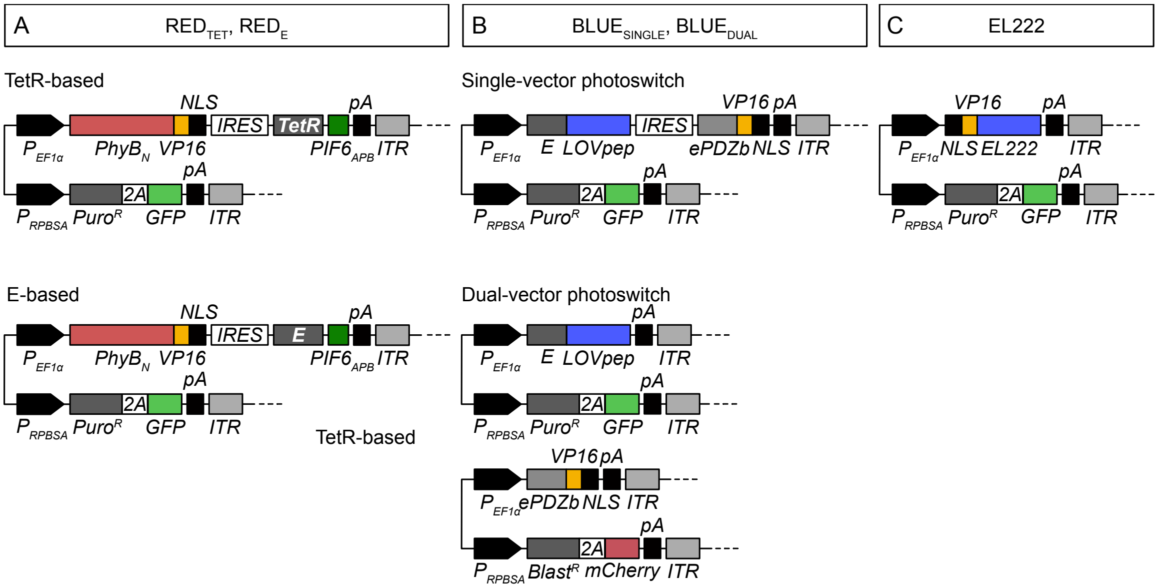

Figure S1. Transposition-competent genetic constructs encoding red and blue light-responsive photoswitches for the optogenetic gene expression devices.

A) RED vectors encoding the *PhyB$_N$/PIF6$_{APB}$* photoswitch either utilizing TetR (RED$_{TET}$), or E (RED$_E$) as the DNA-binding domain.

B) BLUE vectors encoding the *LOVpep/ePDZb* photoswitch either encoded on a single bicistronic vector (BLUE$_{SINGLE}$) with puromycin resistance or on two vectors with additional blasticidin resistance (BLUE$_{DUAL}$).

C) Vector encoding the *EL222-based* photoswitch.

A-C) For abbreviations see Table S1.

Note to Figure S1

Design of the tested optogenetic gene switches

The RED gene switches constitute artificial split transcription factors (TF) based on the red light-induced heterodimerization between the N-terminal fragment of the photoreceptor phytochrome B from *Arabidopsis thaliana (*PhyB$_N$) and a minimized binding sequence derived from the phytochrome-interacting-factor 6 (PIF6$_{APB}$, Figure 1A). Far red light terminates the interaction. Utilizing DNA-binding domains like the TetR repressor protein from *E. coli* and the *Herpes simplex* transactivator VP16, red light reconstitutes a potent TF protein complex in the presence of a suitable chromophore such as phycocyanobilin (PCB)[1]. In combination with an engineered synthetic promoter combining a TATA-containing minimal promoter with DNA sequences specific for the used DNA-

binding domains (see Figure 1E-F), light-induced TF formation initiates transgene expression. We utilized either TetR (binding *tetO* repeats) as the DNA-binding domain as originally published[1,2], or the orthogonal erythromycin-responsive macrolide-inactivating 2'-phosphotransferase I repressor MphR(A), known as E protein specifically binding *etr* repeats, which we also utilized previously in planta[3] (Figure S1A, Supplementary Information), abbreviated as RED_{TET} and RED_{E}, respectively[4]. TetR is the most widely-used DNA-binding domain employed in engineered gene switches and orthogonal alternatives such as E enables a combined use. Similarly, a TULIP-based system, LOVpep/ePDZb, confers induction of a synthetic reporter upon heterodimerization of an engineered LOV2 domain from phototropin 1 of *Avena sativa* and a PDZ domain (Figure 1B)[5,6]. Here, blue light induces the undocking of a C-terminal epitope tag to allow high-affinity binding of ePDZb, leading to TF reconstitution. In both the RED ($PhyB_{N}$/$PIF6_{APB}$) and BLUE (LOVpep/ePDZb) systems, we used internal ribosome entry sites (IRES) in order to translate the two components of the respective optogenetic split TFs from single mRNAs. Since the performance of split TFs depends on the relative concentrations of the two components, we also tested for the BLUE system, whether individual rounds of genomic vector integration of the single switch components may improve the performance due to unique combinations resulting from random and individual genomic insertion events, giving rise to $BLUE_{SINGLE}$ and $BLUE_{DUAL}$, with the expression cassettes combined on a single or separated on individual vectors, respectively (Figure S1B, Supplementary Information). Lastly, in order to completely prevent eventual performance penalties caused by imbalanced expression ratios, we additionally resorted to the EL222 blue light system, which acts as a single molecular component for which photoexcitation directly regulates the DNA-binding affinity (Figure 1C and Figure S1C, Supplementary Information)[7].

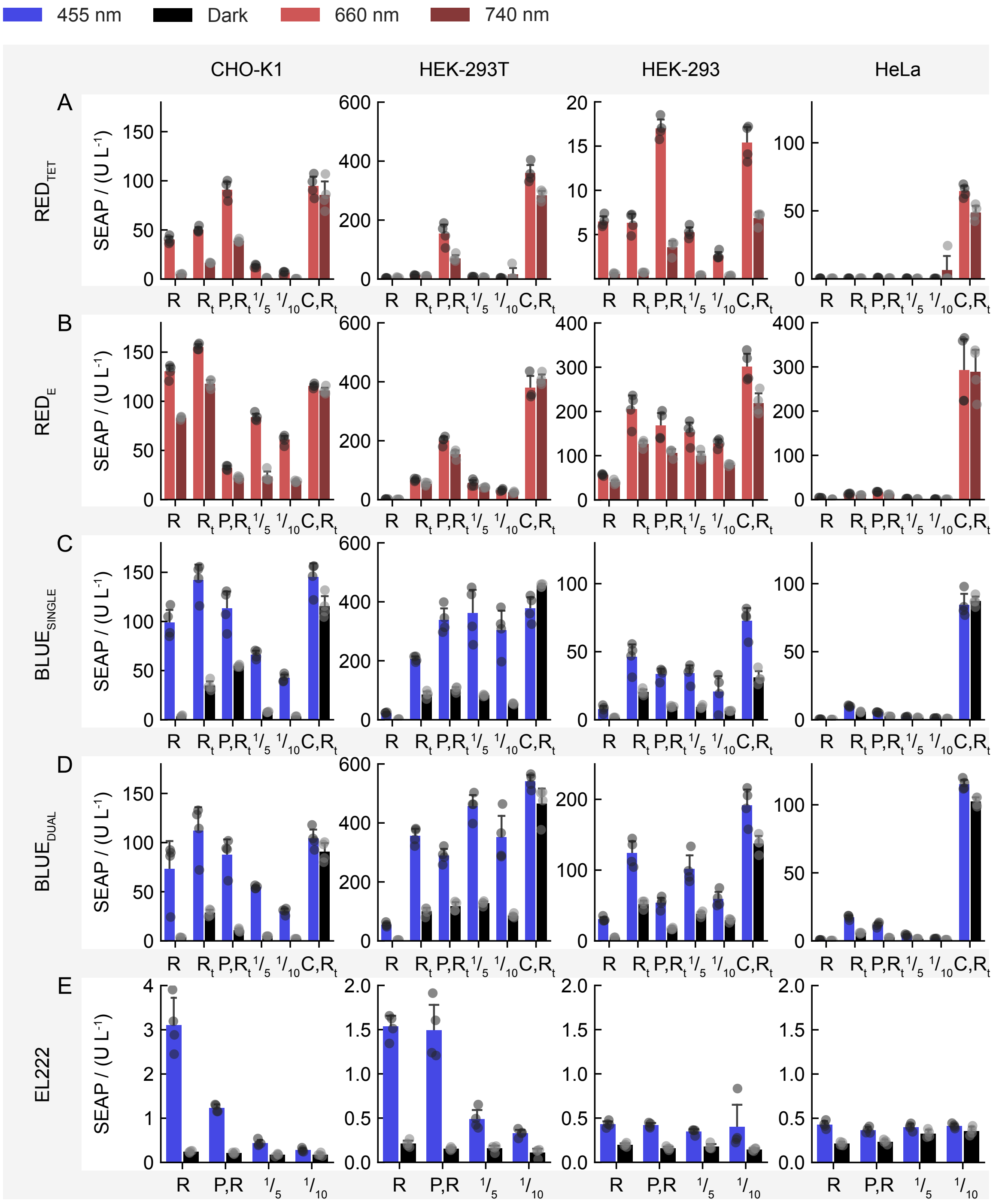

Figure S2. Partial genomic integration of optogenetic gene switches, full dataset. A-E) Cultures of the indicated cell lines represent a mixed population of antibiotic selected cells with individual genomic insertion events of the photoswitch constructs as in Figure S1, Supplementary Information. Additionally, the cultures were transiently transfected with the reporter constructs R or R_t (see Figure 1E), the photoswitch-encoding vector P which

was used for the generation of the culture, a 1/10 or 1/5 reduced amount of R or R_t, or the constitutive activators tTA or eTA in combination with R_t. Cultures were illuminated for 24 h with the indicated light quality before determining the SEAP levels. Mean values of four replicates with one standard deviation are shown except for the 660 nm light sample of HEK-293T with RED_E C,R_t, and the 455 nm light sample of CHO-K1 with $BLUE_{SINGLE}$ and 1/10 R_t, where only three values were used. The data for CHO-K1 cells are also included in Figure 2. Source data are provided with this paper.

Note 1 to Figure S2

For the red/far-red light-responsive system based on PhyB and PIF6, we tested constructs harboring either TetR, or E as the DNA-binding domain, while all remaining constructs utilized E, with the exception of the EL222 system due to its natural DNA-binding capability. For the LOVpep/ePDZb blue light system, we instead questioned whether bicistronic vectors or individual genomic integration and selection events would enhance the chances to isolate well-performing clones. In the initial reports of the RED gene expression system, the promoter activity in the dark state was attenuated by introducing additional spacing between the operator and the promoter, which comes at the cost of lowering the promoter strength in the illuminated state[1]. As we might expect a much-reduced expression strength after integration into the genome, we decided not to apply such attenuating strategies.

Note 2 to Figure S2

The synthetic RPBSA promoter is located downstream of the inducible promoter and is of enhancer-less nature, however, prior to genomic integration, the circular architecture of the plasmid may cause an expression-enhancing effect. A second contribution might arise from the presumably high ratio in copy number of the genomically-encoded photoswitch and the transiently-transfected reporter plasmids. To simulate the effect of genomic integration in the latter case, we also reduced the amount of reporter plasmid DNA in our tests.

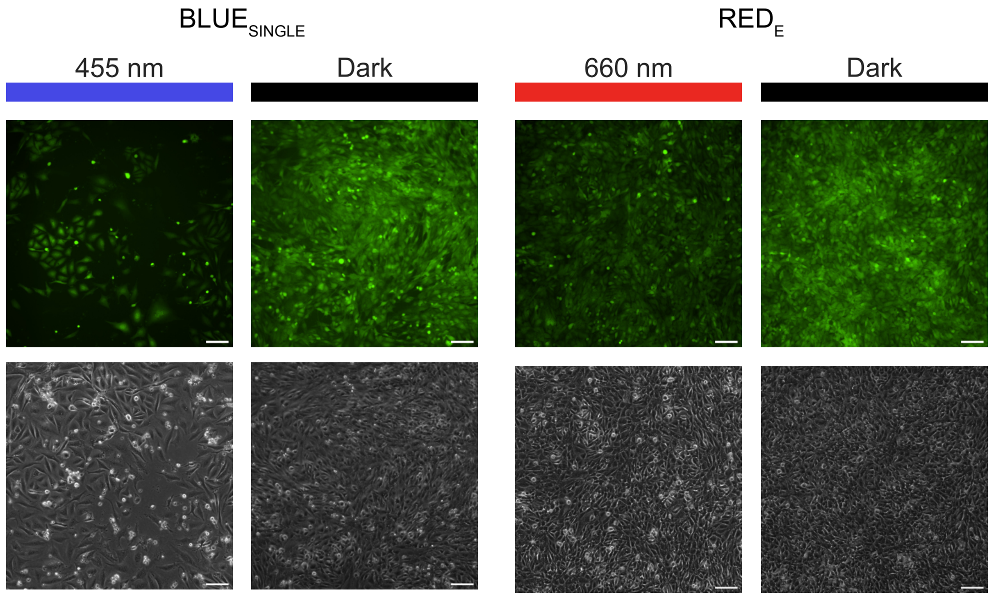

Figure S3. Effects of blue and red light induction of necroptosis in bulk cultures. Induction of necroptosis in CHO-K1 bulk cultures using $BLUE_{SINGLE}$ (left, 10 µmol m^{-2} s^{-1} 455 nm LED light), and RED_E (right, 20 µmol m^{-2} s^{-1} 660 nm LED light) after 24 h. Top, EGFP; bottom, brightfield. Representative images (n = 6 in two independent experiments). Scale bar, 100 µm.

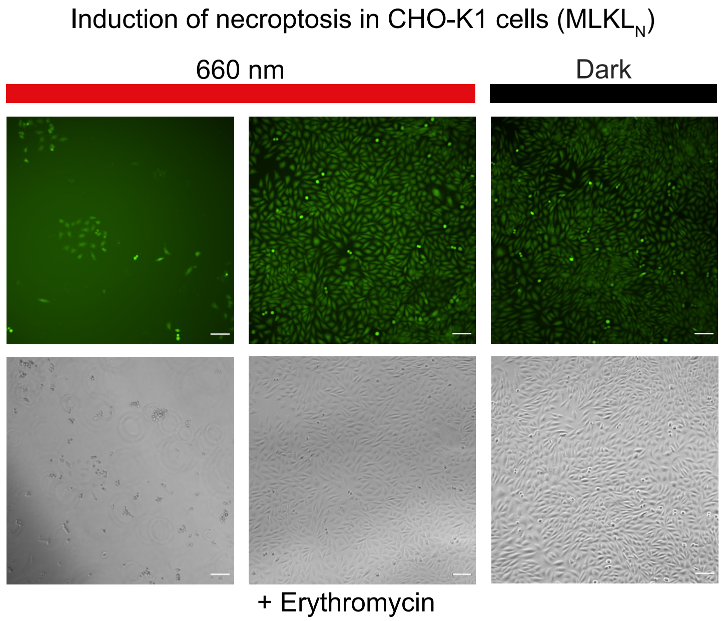

Figure S4. Red light-induction of necroptosis in a selected clone of a CHO-K1 cell line using the RED$_E$ gene switch (Figure S1A) in combination with the *MLKL$_N$*-encoding vector as in Figure 4A. Cells were illuminated with 20 µmol m^{-2} s^{-1} red light (overall LED illumination) or kept in the dark for 24 h followed by microscopic imaging. Top, EGFP; bottom, brightfield. Representative images (n = 6 in two independent experiments). Scale bar, 100 µm.

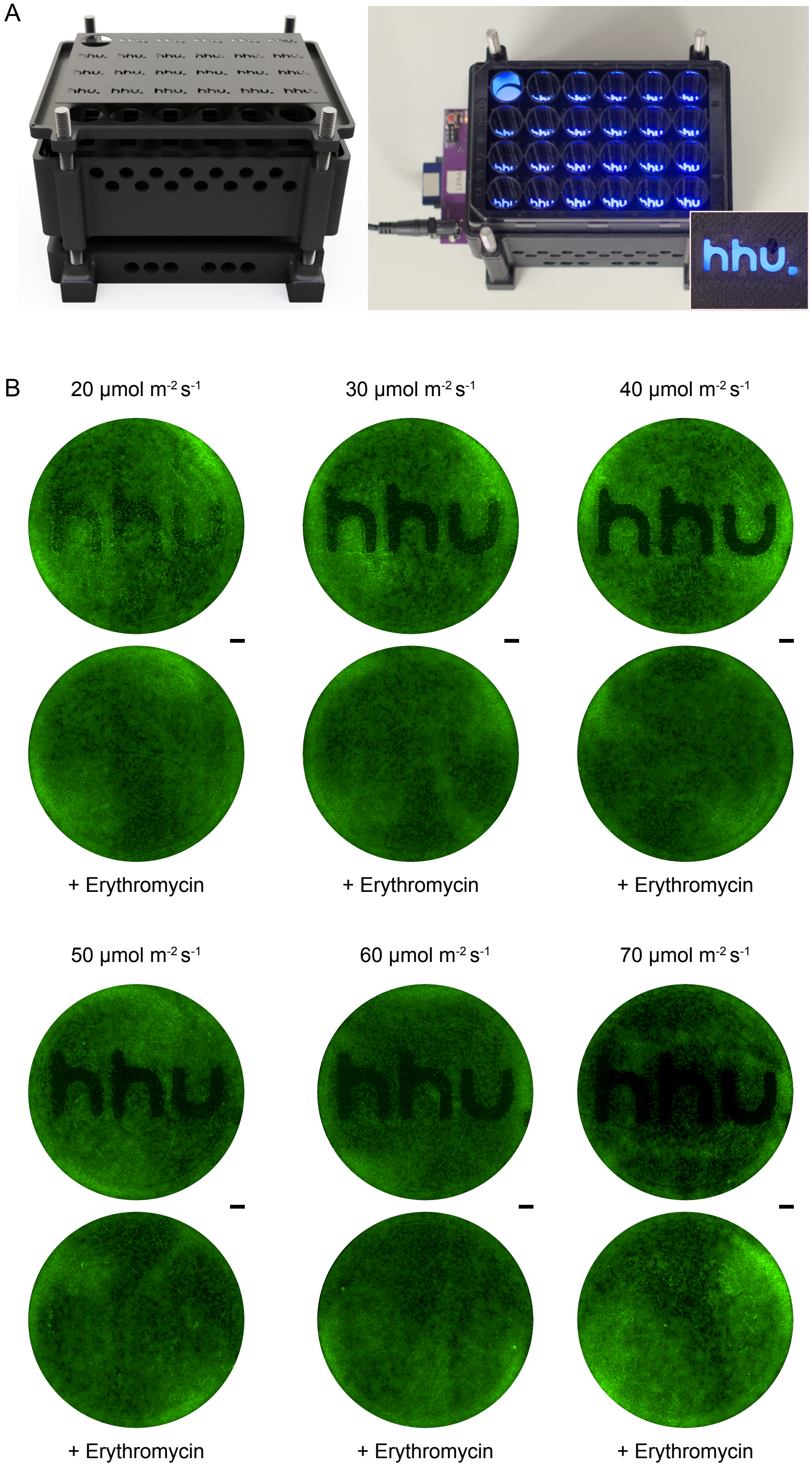
A
B
20 µmol m^{-2} s^{-1}
30 µmol m^{-2} s^{-1}
40 µmol m^{-2} s^{-1}
+ Erythromycin
+ Erythromycin
+ Erythromycin
50 µmol m^{-2} s^{-1}
60 µmol m^{-2} s^{-1}
70 µmol m^{-2} s^{-1}
+ Erythromycin
+ Erythromycin
+ Erythromycin

Figure S5. Spatial induction of necroptosis using a 3D-printed photomask. A) Rendered CAD model of a Light plate apparatus (LPA)[8] chassis with a mounted 3D-printed photomask next to a plate-mounted device equipped with 460 nm LEDs. A magnified mask is shown in the lower right corner. B) CHO-K1^{NecrOpto} cells were illuminated with the indicated light intensities of 460 nm blue light in the center of each well for 24 h through a photomask using the LPA device. The bottom samples show the erythromycin control at identical conditions. The experiment was repeated three times with similar results. Scale bar, 1 mm.

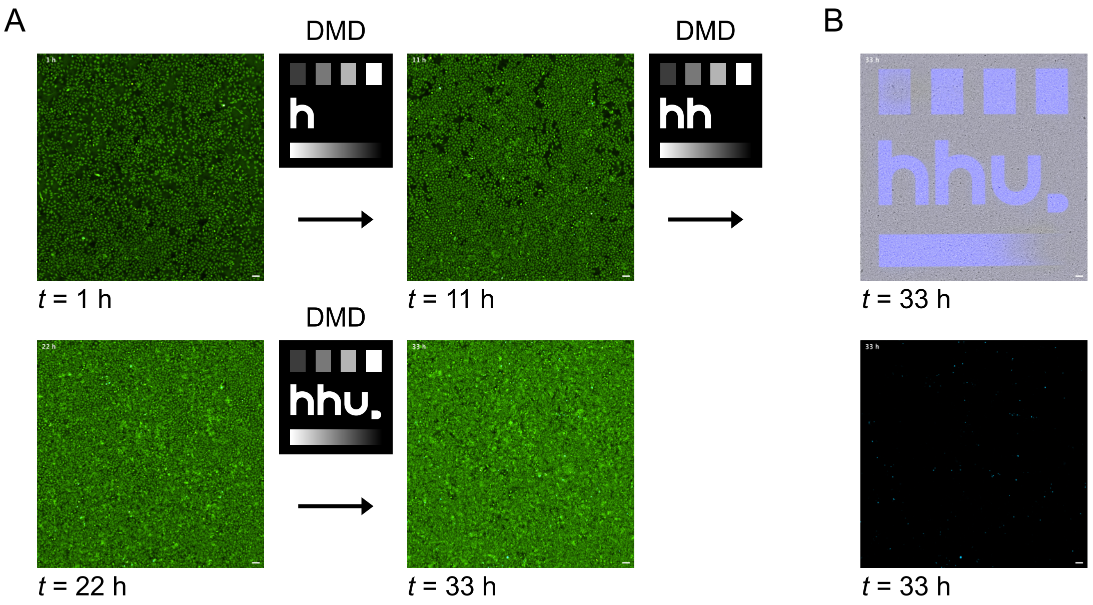

Figure S6. Erythromycin supplementation protects the engineered CHO-K1 cells from inducing necroptosis upon blue light illumination *via* the DMD by displacing the E DNA-binding protein from the DNA operator sequence. A-B) Experiment as in Figure 5B-C but with erythromycin supplementation (2 µg/mL). 8-bit grey shade values are 60, 120, 180, and 255. Linear gradient, 200-0. Scale bar, 100 µm.

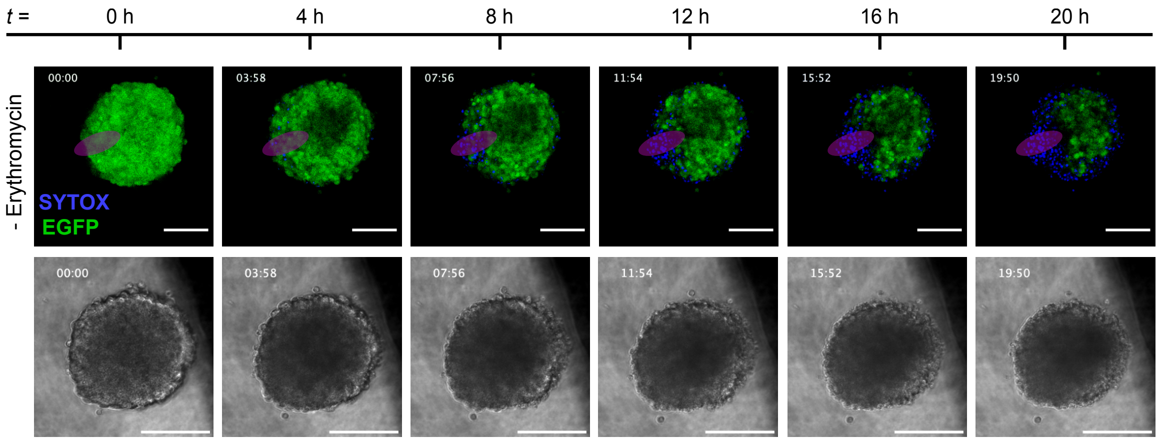

Figure S7. Unspecific induction of necroptosis due to extensive imaging. Experiment as in Figure 5D but with additional brightfield imaging. The CHO-K1^{NecrOpto} spheroid was illuminated in a loop using the confocal laser unit at 488 nm in the ROI (purple) for 30 min with 0.2 a.u., interrupted by brightfield and fluorescence image acquisition in 488 (GFP) and 405 nm (SYTOX) followed by six z-stacks of confocal imaging at 405 and 488 nm. Epifluorescence images are not shown. Maximum z-projections of the confocal images are shown on top, the brightfield images are depicted below. Scale bar, 100 µm.

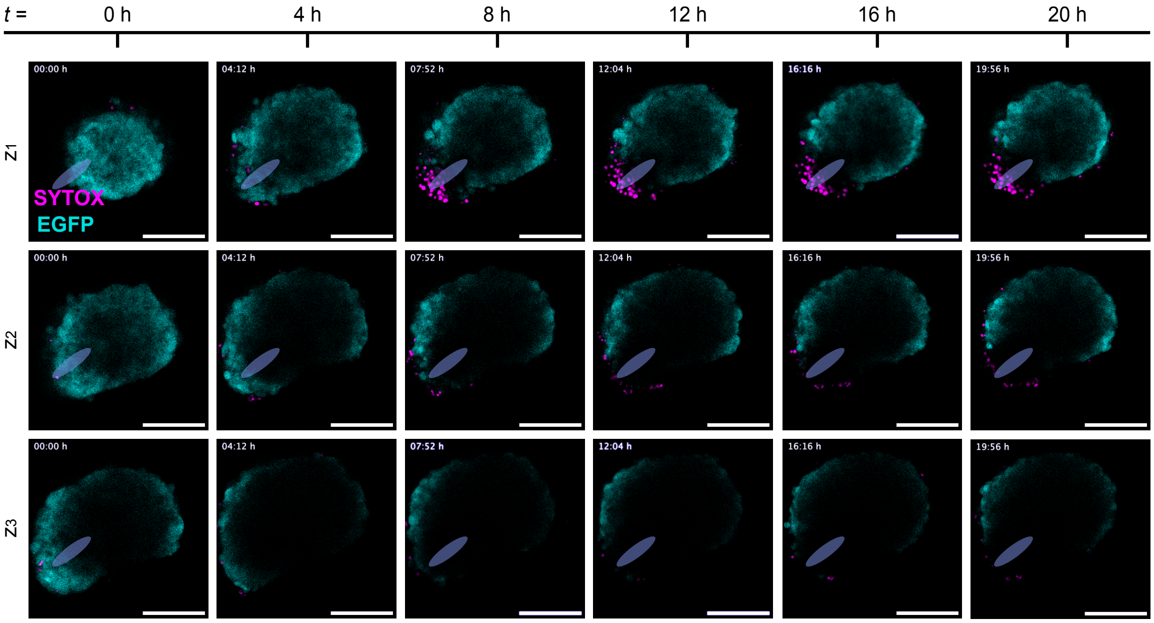

Figure S8. Z-layers of the intensity projection images in Figure 5D. Layers Z1-Z3 are shown with a distance of 19.81 µm between two layers. Scale bar, 100 µm.

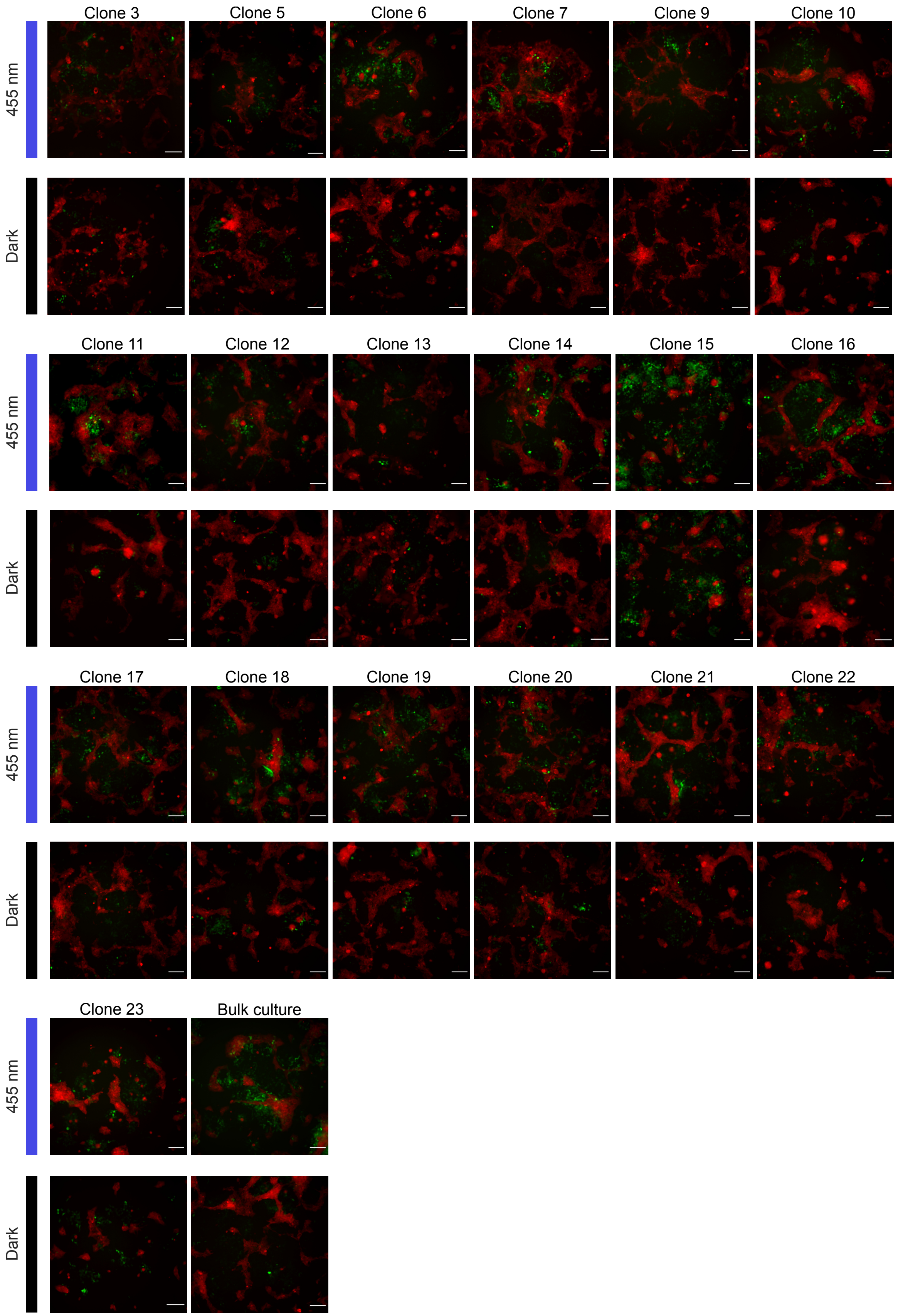
Clone 3
Clone 5
Clone 6
Clone 7
Clone 9
Clone 10
455 nm
Dark
Clone 11
Clone 12
Clone 13
Clone 14
Clone 15
Clone 16
455 nm
Dark
Clone 17
Clone 18
Clone 19
Clone 20
Clone 21
Clone 22
455 nm
Dark
Clone 23
Bulk culture
455 nm
Dark

Figure S9. Profile of randomly selected clones of HEK$^{Cdh3\text{-}OptoWnt}$ cells harboring the BLUE$_{SINGLE}$ gene switch as in Figure 6A. HEK$^{Cdh3\text{-}OptoWnt}$ cells of the indicated clones were mixed with TOP-GFP cells and illuminated with 10 µmol m^{-2} s^{-1} blue light for 24 h prior to imaging, as in Figure 6C. Representative images (n = 2).

Table S1. Plasmids generated and used in this study.

Plasmid	Description	Reference
pCMV(CAT)T7-SB100	***P_{CMV}-Intron$_{SV40}$-SB100X-pA***<br>Constitutive expression vector for the hyperactive evolved Sleeping Beauty transposase SB100X.	Mátés *et al.*,[9] Addgene #34879
pDD001	***P_{SV40}-mCherry-HA-pA***<br>Constitutive expression vector encoding HA-tagged mCherry under the control of a SV40 promoter. The pSAM200 vector backbone was PCR-amplified using oDD001 and oDD002. The mCherry gene was PCR-amplified from pMZ333 using oDD003 and oDD004. The latter war reamplified using oDD003 and oDD005 to attach additional sequence encoding an HA-tag. The two DNA fragments were assembled into the final vector using AQUA Cloning.	This work
pDD104	***ITR-$P_{EF1\alpha}$-PhyB$_N$-VP16-NLS-IRES$_{PV}$-E-PIF6$_{APB}$-pA, P_{RPBSA}-EGFP-P2A-Puro^R-pA-ITR***<br>Bidirectional Sleeping Beauty-compatible vector with constitutive expression of the E-based PhyB$_N$/PIF6$_{APB}$ photoswitch as well as *EGFP* and *Puro^R*. pSBbi-GP was linearized using *Sfi*I, the PhyB$_N$/PIF6$_{APB}$ cassette including a picornaviral IRES was PCR-amplified from pKM300 using the oligonucleotides oDD219 and oDD220, and the products were assembled by Gibson Cloning.	This work
pDD105	***P_{TCE}-SEAP-pA***<br>Reporter plasmid encoding SEAP under the control of the TCE (tetracycline response element and minimal CMV-promoter enhanced) promoter, containing 7 tetO repeats. P_{TCE} was excised from pSBtet-GP using *Nco*I and *Pst*I and Gibson-cloned into pKM006 which was PCR-amplified using the oligonucleotides oDD221 and oDD222.	Jang *et at.*,[10]
pDD106	***ITR-$P_{EF1\alpha}$-PhyB$_N$-VP16-NLS-IRES$_{PV}$-TetR-PIF6$_{APB}$-pA, P_{RPBSA}-EGFP-P2A-Puro^R-pA-ITR***<br>Bidirectional Sleeping Beauty-compatible vector with constitutive expression of the TetR-based PhyB$_N$/PIF6$_{APB}$ photoswitch as well as *EGFP* and *Puro^R*. pSBbi-GP was linearized using *Sfi*I, the PhyB$_N$/PIF6$_{APB}$ cassette including a picornaviral *IRES* was PCR-amplified from pKM022 using the oligonucleotides oDD223 and oDD224, and the products were assembled by Gibson Cloning.	This work
pDD107	***ITR-$P_{EF1\alpha}$-E-LOVpep-IRES$_{PV}$-ePDZb-VP16-NLS-pA, P_{RPBSA}-EGFP-P2A-Puro^R-pA-ITR***<br>Bidirectional Sleeping Beauty-compatible vector with constitutive expression of the E-based LOVpep/ePDZb photoswitch as well as *EGFP and Puro^R.* The E-protein-encoding gene fused to *LOVpep* was PCR-amplified from pKM549 using the oligonucleotides oDD225 and oDD234. IRES$_{PV}$-ePDZb-VP16-NLS was amplified from pKM516 using oDD226 and oDD233. The fragments were fused by PCR using oDD225 and oDD226, and ligated into pSBbi-GP using *Sfi*I.	This work
pDD108	***ITR-$P_{EF1\alpha}$-E-LOV$_{pep}$-pA, P_{RPBSA}-EGFP-P2A-Puro^R-pA-ITR***<br>Bidirectional Sleeping Beauty-compatible vector with constitutive expression of the E DNA-binding protein-coding gene fused to LOV$_{pep}$. Constitutive expression of *EGFP* and *Puro^R*. Sequence encoding E-LOVpep was PCR-amplified from pKM549 using the oligonucleotides oDD228 and oDD229 and Gibson-cloned into *Sfi*I-digested pSBbi-GP.	This work
pDD109	***ITR-$P_{EF1\alpha}$-ePDZB-VP16-NLS-pA, P_{RPBSA}-dTomato-P2A-Hyg^R-pA-ITR***<br>Bidirectional Sleeping Beauty-compatible vector with constitutive expression of *ePDZB* fused to *VP16* and an *NLS*. Constitutive expression of dTomato and *Hyg^R*. ePDZB-	This work

	VP16-NLS was PCR- amplified from pKM516 using oDD227 and oDD230 and Gibson-cloned into *Sfi*I-digested pSBbi-RP.	
pDD110	***ITR-$P_{EF1\alpha}$-VP16-EL222-pA, P_{RPBSA}-EGFP-P2A-$Puro^R$-pA-ITR***<br>Bidirectional Sleeping Beauty-compatible vector with constitutive expression of the *EL222* photoswitch fused to *VP16*. Constitutive expression of *EGFP* and *$Puro^R$*. Sequence encoding EL222 was PCR-amplified from pIRES-Puro-VP-EL222 using the oligonucleotides oDD231 and oDD232 and Gibson-cloned into *Sfi*I-digested pSBbi-GP.	This work
pDD115	***ITR-$P_{EF1\alpha}$-ePDZB-VP16-NLS-pA, P_{RPBSA}-dTomato-P2A-$Blast^R$-pA-ITR***<br>Bidirectional Sleeping Beauty-compatible vector with constitutive expression of *ePDZB* fused to *VP16* and an *NLS*. Constitutive expression of *dTomato* and *$Blast^R$*. *$Blast^R$* was PCR-amplified from pWW29 using oDD253 and oDD254. The vector backbone was PCR-amplified from pDD109 using oDD251 and oDD252. Both fragments were assembled using Gibson Cloning.	This work
pDD123	***ITR-etr_8-P_{min}-SEAP-pA, P_{RPBSA}-Hyg^R-pA-ITR***<br>Bidirectional Sleeping Beauty-compatible vector containing an E-responsive inducible promoter controlling the expression of SEAP. Constitutive expression of *Hyg^R*. SEAP was PCR-amplified from pKM081 using oDD284 and oDD285. The vector backbone was PCR-amplified from pDD203 using oDD286 and oDD287. Both fragments were assembled using Gibson Cloning.	This work
pDD203	***ITR-etr8-P_{min}-mWnt3a-$IRES_{PV}$-mCherry-HA-pA-P_{RPBSA}-Hyg^R-pA-ITR***<br>Sleeping Beauty-compatible vector containing an E-regulated inducible promoter controlling the expression of the mouse *Wnt3a* gene and *mCherry*. Constitutive expression of *Hyg^R*. The *mWnt3a* gene was PCR-amplified from pENTR-mCherry-2A-Wnt3a using the oligonucleotides oDD196 and oDD197. The poliviral *IRES* sequence was amplified from pKM022 using oDD198 and oDD199. *mCherry-HA-pA* was amplified from pDD001 using oDD300 and oDD301. The P_{RPBSA}-controlled constitutive expression cassette for *Hyg^R* was amplified from pSBbi-Hyg together with the vector backbone using oDD194 and oDD195. The E-responsive promoter containing 8 repeats of the E-binding operator together with P_{min} was excised from pKM081 using *Hind*III and *Xba*I. All fragments were simultaneously assembled into the final vector using Gibson Cloning.	This work
pDD206	***ITR-$P_{EF1\alpha}$-$PhyB_N$-VP16-NLS-$IRES_{PV}$-TetR-$PIF6_{APB}$-pA, P_{RPBSA}-$Puro^R$-pA-ITR***<br>Bidirectional Sleeping Beauty-compatible vector for constitutive expression of the TetR-based $PhyB_N$/$PIF6_{APB}$ photoswitch and *$Puro^R$*. The EGFP- and T2A-encoding sequences were deleted from plasmid pDD106 by PCR amplifications using oDD314/oDD313 and oDD312/oDD315 followed by Gibson assembly.	This work
pDD207	***ITR-$P_{EF1\alpha}$-E-LOVpep-$IRES_{PV}$-ePDZb-VP16-NLS-pA, P_{RPBSA}-$Puro^R$-pA-ITR***<br>Bidirectional Sleeping Beauty-compatible vector with constitutive expression of the E-based LOVpep/ePDZb photoswitch as well as *$Puro^R$.* The EGFP-coding sequence was deleted from pDD107 by PCR-amplification using the oligonucleotide-pairs oDD312/oDD315 and oDD313/oDD314. The two fragments were assembled using Gibson Cloning.	This work
pDD208	***ITR-$P_{EF1\alpha}$-$PhyB_N$-VP16-NLS-$IRES_{PV}$-E-$PIF6_{APB}$-pA, P_{RPBSA}-$Puro^R$-pA-ITR***<br>Bidirectional Sleeping Beauty-compatible vector with constitutive expression of the TetR-based $PhyB_N$/$PIF6_{APB}$ photoswitch and *$Puro^R$*. The EGFP-coding sequence was deleted from pDD106 by PCR-amplification using the oligonucleotide-pairs oDD312/oDD315 and oDD313/oDD314. The two fragments were assembled using Gibson Cloning.	This work

pDD218	***P_{CMV}-tetO$_2$-mCherry-2A-mWnt3a***	This work
pENTR-mCherry-2A-Wnt3a	TetR-repressible CMV promoter-controlled expression plasmid encoding the red fluorescent protein mCherry and mouse WNT3A.	Glykofrydis *et al.,*[11]
pIRES-Puro-VP-EL222	***P_{CMV}-NLS-VP16-EL222-IRES$_{EMCV}$-Puro^R-pA***<br>Constitutive bicistronic CMV promoter-driven expression vector encoding NLS-VP16-EL222 and Puro^R.	Motta-Mena et al., [7]
pKM006	***tetO$_{13}$-422 bp-P_{min}-SEAP-pA***<br>SEAP reporter plasmid harboring a modified P_{tet} with a 422-bp spacer between the 13mer *tetO* operator and the minimal promoter.	Müller *et al.*,[1]
pKM022	***P_{SV40}-PhyB$_N$-VP16-NLS-IRES$_{PV}$-TetR-PIF6$_{APB}$-pA***<br>Bicistronic expression vector encoding the PhyB$_N$/PIF6$_{APB}$ system using TetR as DNA-binding domain under the control of an SV40 promoter.	Müller *et al.*,[1]
pKM081	***etr8-P_{min}-SEAP-pA***<br>SEAP reporter plasmid harboring a modified P_{etr}.	Müller *et al.*,[12]
pKM082	**etr8-386bp-P_{min}-SEAP-pA**<br>SEAP reporter plasmid derivative of pKM081 containing a 386 bp spacer between the etr operator repeats and P_{min}.	Müller *et al.*,[3]
pKM300	***P_{SV40}-PhyB$_N$-VP16-NLS-IRES$_{PV}$-E-PIF6$_{APB}$-pA***<br>Bicistronic expression vector encoding the PhyB$_N$/PIF6$_{APB}$ system using E as DNA-binding domain under the control of an SV40 promoter.	Müller *et al.*,[3]
pKM516	***P_{SV40}-Gal4BD-LOVpep-IRES$_{PV}$-ePDZb-VP16-NLS-pA***<br>Bicistronic vector encoding Gal4BD-LOVpep and ePDZb-VP16-NLS under control of P_{SV40}.	Müller *et al.*,[5]
pKM549	**P_{SV40}-E-LOVpep-pA**<br>Constitutive SV40 promoter-driven expression vector encoding E-LOVpep.	Unpublished
pKM583	***(C120)$_5$-P_{min}-SEAP-pA***<br>SEAP reporter plasmid harboring five repeats of the EL222-specific DNA operator sequence (C120) upstream of the cytomegalovirus minimal promoter (P_{min}).	Unpublished
pMN015	***ITR-etr8-P_{min}-MLKL$_N$-T2A-mCherry-pA-P_{RPBSA}-HygR-pA-ITR***<br>The gene encoding the 178 N-terminal residues of the human Mixed Lineage Kinase Domain-Like protein (MLKL$_N$) was synthesized and PCR-amplified using the oligonucleotides oMN002 and oMN003. The sequence encoding mCherry was amplified from pDD001 using oMN004 and oMN005. The two fragments were Gibson-cloned into *Sal*I and *Xho*I-digested pDD123. The sequence encoding the T2A peptide resulted from 5' primer extensions.	This work
pMZ333	Unpublished vector encoding the red fluorescent protein mCherry.	Unpublished
pSBbi-GP	***ITR-$P_{EF1\alpha}$-pA, P_{RPBSA}-EGFP-P2A-Puro^R-pA-ITR***<br>Bidirectional empty Sleeping Beauty-compatible vector with constitutive expression of *EGFP* and *Puro^R*.	Kowarz *et al.*,[13], Addgene #60511
pSAM200	***P_{SV40}-TetR-VP16-pA***<br>Constitutive expression vector encoding tTA (TetR-VP16).	Fussenegger *et al.,*[2]
pSBbi-Hyg	***ITR-$P_{EF1\alpha}$-pA, P_{RPBSA}-Hyg^R-pA-ITR***<br>Bidirectional empty Sleeping Beauty-compatible vector with constitutive expression of *Hyg^R*.	Kowarz *et al.*,[13], Addgene #60524

pSBbi-RP	***ITR-P$_{EF1\alpha}$-pA, P$_{RPBSA}$-dTomato-P2A-Hyg^{R}-pA-ITR***<br>Bidirectional empty Sleeping Beauty-compatible vector with constitutive expression of *dTomato* and *Puro^{R}*.	Kowarz *et al.*,[13], Addgene #60513
pSBtet-GP	***ITR-P$_{TCE}$-luc2-pA-P$_{RPBSA}$-EGFP-P2A-rtTA-P2A-Puro^{R}-pA-ITR***<br>Sleeping Beauty-compatible vector with tetracycline-inducible expression of the luc2 variant firefly luciferase controlled by P$_{TCE}$ (see pDD105) and constitutive expression of *EGFP, rtTA*, and *Puro^{R}*.	Kowarz *et al.*,[13], Addgene #60495
pWW29	***P$_{EF1\alpha}$ -E-pA***<br>Vector encoding the erythromycin repressor protein E under control of P$_{EF1\alpha}$ and containing the *Blast^{R}* gene.	Weber *et al.*,[4]
pWW35	***P$_{SV40}$-E-VP16-pA***<br>Constitutive expression vector encoding eTA (E-VP16).	Weber *et al.*,[4]

Abbreviations:

Blast^{R}, blasticidin-S deaminase - bsd;

(C120)$_5$, EL222 operator sequence;

E, E-protein - 2-phosphotransferase I (mph(A)) from *E. coli;*

EL222, *Erythrobacter litoralis*-derived blue light-regulated transcription factor;

ePDZb, low affinity Erbin PDZ domain variant;

etr$_8$, erythromycin repressor operator repeats;

Gal4BD, DNA-binding domain of Ga4;

HA, human influenza hemagglutinin tag;

Hyg^{R}, aminoglycoside O-phosphotransferase - APH(4)-Ia;

IRES$_{EMCV}$, internal ribosome entry site derived from the encephalomyocarditis virus

IRES$_{PV}$, Polioviral internal ribosome entry site;

ITR, Sleeping Beauty inverted tandem repeats;

LOVpep, truncated *As*LOV2 of phototrophin 1 [p.404-543, p.T406A,T407A,I532A] with c-terminal fusion to epitope peptide "KAVDTWV";

luc2, mammalian cell codon-optimazed Firefly luciferase variant without cryptic transcription factor binding sites;

MLKL$_N$, sequence encoding the 178 N-terminal residues of the Mixed Lineage Kinase Domain Like protein;

NLS, nuclear localization signal of SV40;

P2A, self-cleaving peptide derived from porcine teschovirus-1 2A;

pA, polyadenylation signal;

P$_{CMV}$, cytomegalovirus promoter;

P$_{EF1\alpha}$, constitutive human elongation factor-1 alpha promoter;

PhyB, Arabidopsis thaliana Phytochrome B;

PIF6, Arabidopsis thaliana phytochrome-interacting factor 6;

P$_{RPBSA}$, constitutive synthetic enhancer-less promoter derived from the *RPL13a* promoter fused to a region of the *RPL41* gene;

P$_{TCE}$, inducible tetracycline response element and minimal CMV-promoter enhanced promoter containing seven tetO repeats;

Puro^{R}, puromycin N-acetyltransferase mediating resistance to puromycin in mammalian cells;

rtTA, reversed tetracycline transactivator;

SEAP, human placental secreted alkaline phosphatase;

SB100X, hyperactive Sleeping Beauty transposase;

T2A, self-cleaving peptide derived from thosea asigna virus 2A;

TetR, tetracycline repressor protein;

VP16, transactivation domain of *Herpes simplex* virus protein vmw65.

Table S2. Oligonucleotides used in this study.

Name	Sequence (5' → 3')	Reference
oDD001	TAATCTAGAGTCGACCTGCAGC	This work
oDD002	GGTGGCGCCTCCTGAATTCGCGGCCGCAATTC	This work
oDD003	GCCGCGAATTCAGGAGGCGCCACCATGGTGAGCAAGGGCGAG	This work
oDD004	AGCGTAATCCGGTACGTCGTAAGGGTAGGATCCGCTGCCCTTGTACAGCTCGTCCATGC	This work
oDD005	GGGCTGCAGGTCGACTCTAGATTAAGCGTAATCCGGTACGTCG	This work
oDD145	GTCTCCTCTGACTTCAACAGCG	This work
oDD146	ACCACCCTGTTGCTGTAGCCAA	This work
oDD194	ATTATGATCCTGCCTCCTAGGCGAGACCCTGTCTC	This work
oDD195	TATTCAATCGTTTAAACTTCGAAGCTTCTAGATAGCGGACCCCTTACC	This work
oDD196	ATTCGAGCTCGCCCGGGGATCCTCTAGAGTCAGCTTCCACCATGGCTCCTCTCGGATACC	This work
oDD197	TGGGCTGCAGGTCGACCTACTTGCAGGTGTGCACG	This work
oDD198	GCACACCTGCAAGTAGGTCGACCTGCAGCCCAAG	This work
oDD199	TTGCTCACCATGGTGGGAATTCGCGGCCGCAATC	This work
oDD219	GTGAAAACTACCCCAAGCTGGCCTCTGAGGCCACCATGGTTTCCGGAGTC	This work
oDD220	AGAATTGATCCCCAAGCTTGGCCTGACAGGCCTTTAAGCGTAATCTGGAACATCGTATG	This work
oDD221	ACCCCTTACCGAAACATCGCCGCATTCTGCAGGATATCGTCGACGACGTCAGG	This work
oDD222	TCCTACCCTCGAAAGGCCTCTGAGGCCACCATGCTGCTGCTGCTGCTG	This work
oDD223	AATTGATCCCCAAGCTTGGCCTGACGGCCTGACAGGCCTTAAGCGTAATCTGGAACATCG	This work
oDD224	GTCGTGAAAACTACCCCAAGCTGGCCTCTGGCCTCTGAGGCCACCATGGTTTCCGG	This work
oDD225	AAAGGCCTCTGAGGCCACCATGCCCCGCC	This work
oDD226	AATGGCCAGACAGGCCTCACACCTTCCGCTTTTTC	This work
oDD233	GCAGTCATGCTGGCTAAG	This work
oDD234	GATCCTTACACCCAGGTATCCAC	This work
oDD227	AATTGATCCCCAAGCTTGGCCTGACGGCCTGACAGGCCTCACACCTTCCGCTTTTTCTTG	This work
oDD228	GTGAAAACTACCCCAAGCTGGCCTCTGGGCCTCTGAGGCCCCACCATGCCCCGCC	This work
oDD229	AGAATTGATCCCCAAGCTTGGCCTGACGGCCTGACAGGCCTTACACCCAGGTATCCACCG	This work
oDD230	GTGAAAACTACCCCAAGCTGGCCTCTGGGCCTCTGAGGCCCCACCATGCCAGAACTTGG	This work
oDD231	GAGAATTGATCCCCAAGCTTGGCCTGACGGCCTGACAGGCCTTAGATTCCGGCTTCGACG	This work
oDD232	GAAAACTACCCCAAGCTGGCCTCTGGGCCTCTGAGGCCACCATGGGCCCTAAAAAGAAGC	This work
oDD253	GTGACGTCGAGGAGAATCCTGGCCCCATGGCCAAGCCTTTGTCTCAAGAAG	This work
oDD254	GAGTGAATTCACGACAGGCCTTCGAATTAGCCCTCCCACACATAACCAGA	This work
oDD251	CCTCTGGTTATGTGTGGGAGGGCTAATTCGAAGGCCTGTCGTGAATTC	This work
oDD252	TCTTCTTGAGACAAAGGCTTGGCCATGGGGCCAGGATTCTCCTCG	This work
oDD284	CTAGAGTCAGCTTCTCGAGCCACCATGCTGCTGCTGCTGC	This work
oDD285	GGCTGCAGGTCGACGCATGCTTAACCCGGGTGCGC	This work
oDD286	GCAGCAGCAGCAGCATGGTGGCTCGAGAAGCTGACTCTAGAGGATCCC	This work
oDD287	GCCGCGCACCCGGGTTAAGCATGCGTCGACCTGCAGCCC	This work
oDD300	TTGCGGCCGCGAATTCCCACCATGGTGAGCAAGG	This work
oDD301	ACAGGGTCTCGCCTAGGAGGCAGGATCATAATCAGCC	This work
oDD312	CCTCTGCACCTGAGGCCACCATGACCGAGTACAAGCCCAC	This work
oDD313	ACTCGGTCATGGTGGCCTCAGGTGCAGAGGTTTCTACAGGG	This work
oDD314	TTCATTCAGCTCCGGTTCCC	This work
oDD315	TTTTGCACAACATGGGGGATC	This work
oDD337	CATGCACCTCAAGTGCAAATGC	This work

oDD338	TGAGGAAATCCCCGATGGTG	This work
oMN002	CGGGGATCCTCTAGAGTCAGCTTCTCGAGCCACCATGGAGAATCTCAAAC	This work
oMN003	CCGCATGTTAGAAGACTTCCTCTGCCCTCTCCTCCGGACCCTTGTCTGAGAGTCTCCTTTAT TTCTTTC	This work
oMN004	GGCAGAGGAAGTCTTCTAACATGCGGTGACGTGGAGGAGAATCCTGGCCCAGTGAGCAAG GGCGAGGAG	This work
oMN005	CTGGATCGAAGCTTGGGCTGCAGGTCGACTTACTTGTACAGCTCGTCCATGC	This work

Supplementary References

1. Müller, K. *et al.* A red/far-red light-responsive bi-stable toggle switch to control gene expression in mammalian cells. *Nucleic Acids Res.* **41**, e77 (2013).
2. Fussenegger, M., Moser, S., Mazur, X. & Bailey, J. E. Autoregulated multicistronic expression vectors provide one-step cloning of regulated product gene expression in mammalian cells. *Biotechnol. Prog.* **13**, 733–740 (1997).
3. Müller, K. *et al.* A red light-controlled synthetic gene expression switch for plant systems. *Mol. Biosyst.* **10**, 1679–1688 (2014).
4. Weber, W. *et al.* Macrolide-based transgene control in mammalian cells and mice. *Nat. Biotechnol.* **20**, 901–907 (2002).
5. Müller, K., Engesser, R., Timmer, J., Zurbriggen, M. D. & Weber, W. Orthogonal optogenetic triple-gene control in Mammalian cells. *ACS Synth. Biol.* **3**, 796–801 (2014).
6. Strickland, D. *et al.* TULIPs: tunable, light-controlled interacting protein tags for cell biology. *Nat. Methods* **9**, 379–384 (2012).
7. Motta-Mena, L. B. *et al.* An optogenetic gene expression system with rapid activation and deactivation kinetics. *Nat. Chem. Biol.* **10**, 196–202 (2014).
8. Gerhardt, K. P. *et al.* An open-hardware platform for optogenetics and photobiology. *Sci. Rep.* **6**, 35363 (2016).
9. Mátés, L. *et al.* Molecular evolution of a novel hyperactive Sleeping Beauty transposase enables robust stable gene transfer in vertebrates. *Nat. Genet.* **41**, 753–761 (2009).
10. Jang, J. *et al.* Engineering of bidirectional, cyanobacteriochrome-based light-inducible dimers (BICYCL)s. *Nat. Methods* **20**, 432–441 (2023).
11. Glykofrydis, F., Cachat, E., Berzanskyte, I., Dzierzak, E. & Davies, J. A. Bioengineering Self-Organizing Signaling Centers to Control Embryoid Body Pattern Elaboration. *ACS Synth. Biol.* **10**, 1465–1480 (2021).
12. Müller, K. *et al.* Multi-chromatic control of mammalian gene expression and signaling. *Nucleic Acids Res.* **41**, e124 (2013).
13. Kowarz, E., Löscher, D. & Marschalek, R. Optimized Sleeping Beauty transposons rapidly generate stable transgenic cell lines. *Biotechnol. J.* **10**, 647–653 (2015).
