# Supplementary material for: Genetically-stable engineered optogenetic gene switches modulate spatial cell morphogenesis in two- and three-dimensional tissue cultures
Source: Nat Commun. 2024 Dec 2;15:10470. doi: 10.1038/s41467-024-54350-7 (PMC11612184; doi:10.1038/s41467-024-54350-7)
Supplement: Supplementary file 2 — Description of Additional Supplementary Files [file 41467_2024_54350_MOESM2_ESM.docx]

Description of Additional Supplementary Files

**File Name:** Supplementary Movie 1

**Description:** Live imaging of spatial, dynamic, and quantitative optogenetic control of necroptosis using the digital mirror device in Figure 5B-C. Left, overlay of EGFP, mCherry, and SYTOX Blue signals. Right, overlay of brightfield and blue light projection pattern. See Figure 5B-C for additional information and Figure S6 and Movie S2, Supplementary Information for controls. Scale bar, 100 µm.

**File Name:** Supplementary Movie 2

**Description:** Erythromycin control of spatial, dynamic, and quantitative optogenetic ontrol of necroptosis using the digital mirror device in Figure S6. Cultures were supplemented with 2 µg/mL erythromycin. Left, overlay of EGFP, mCherry, and SYTOX Blue signals. Right, overlay of brightfield and blue light projection pattern. See Figure S6 for additional information and Figure 5B-C and Movie S2 corresponding experiments without erythromycin protection. Scale bar, 100 µm.

**File Name:** Supplementary Movie 3

**Description:** Spatial induction of necroptosis in a 3D culture using 488 nm laser excitation over a period of 20 h. Video of experiment in Figure 5D. Scale bar, 100 µm.

**File Name:** Supplementary Movie 4

**Description:** Erythromycin control of spatial induction of necroptosis in a 3D culture using 488 nm laser excitation over a period of 20 h. Video of experiment in Figure 5D. Scale bar, 100 µm.

**File Name:** Supplementary Movie 5

**Description:** Unspecific induction of necroptosis due to extensive imaging. Video of experiment in Figure S7. Scale bar, 100 µm.

**File Name:** Supplementary Data 1

**Description:** Zip archive containing GenBank plasmid maps of the constructs: pDD104, pDD106, pDD107, pDD110, pDD115, pDD123, pDD203, pDD206, pDD207, pDD218, MN015.
