# Supplementary material for: Genetically-stable engineered optogenetic gene switches modulate spatial cell morphogenesis in two- and three-dimensional tissue cultures
Source: Nat Commun. 2024 Dec 2;15:10470. doi: 10.1038/s41467-024-54350-7 (PMC11612184; doi:10.1038/s41467-024-54350-7)
Supplement: Supplementary file 9 — Reporting Summary [file 41467_2024_54350_MOESM9_ESM.pdf]

nature portfolio

Corresponding author(s): Mustafa Khammash, Matias Zurbriggen

Last updated by author(s): Oct 10, 2024

Reporting Summary

Nature Portfolio wishes to improve the reproducibility of the work that we publish. This form provides structure for consistency and transparency in reporting. For further information on Nature Portfolio policies, see our Editorial Policies and the Editorial Policy Checklist.

Statistics

For all statistical analyses, confirm that the following items are present in the figure legend, table legend, main text, or Methods section.

n/a	Confirmed	
☐	☒	The exact sample size (n) for each experimental group/condition, given as a discrete number and unit of measurement
☐	☒	A statement on whether measurements were taken from distinct samples or whether the same sample was measured repeatedly
☐	☒	The statistical test(s) used AND whether they are one- or two-sided<br>*Only common tests should be described solely by name; describe more complex techniques in the Methods section.*
☒	☐	A description of all covariates tested
☒	☐	A description of any assumptions or corrections, such as tests of normality and adjustment for multiple comparisons
☐	☒	A full description of the statistical parameters including central tendency (e.g. means) or other basic estimates (e.g. regression coefficient) AND variation (e.g. standard deviation) or associated estimates of uncertainty (e.g. confidence intervals)
☐	☒	For null hypothesis testing, the test statistic (e.g. F, t, r) with confidence intervals, effect sizes, degrees of freedom and P value noted<br>*Give P values as exact values whenever suitable.*
☒	☐	For Bayesian analysis, information on the choice of priors and Markov chain Monte Carlo settings
☒	☐	For hierarchical and complex designs, identification of the appropriate level for tests and full reporting of outcomes
☒	☐	Estimates of effect sizes (e.g. Cohen's d, Pearson's r), indicating how they were calculated

Our web collection on statistics for biologists contains articles on many of the points above.

Software and code

Policy information about availability of computer code

Data collection	- For SEAP quantification, the software for data acquisition with plate readers MikroWin (version 5.18, Berthold) and MARS Data Analysis (version 3.31, BMG Labtech) were used.<br>- For RT-qPCR, Applied Biosystems® Real-Time PCR Software was used.<br>- For microscopic imaging experiments, Nikon NIS-Elements AR5.11.03 was used.<br>- For programming the Light plate apparatus (LPA) illumination device, Iris 1.0.0 (https://taborlab.github.io/Iris/) was used.<br>- For DMD pattern projection, DLP LightCrafter Display EVM GUI (Texas Instruments) was used.<br>- For data acquisition using the light-sheet microscope, µManager (built 20230613) was used together with Python 3.9.7 and pycromanager version 0.27.5. For continuous illumination using the 488 nm laser, Omicron Control Center Software (OCC, OMICRON electronics GmbH, Austria) version 3.9.28 was used.

Data analysis	- Microscopic images were adjusted for brightness and contrast settings using ImageJ 2.3.0/1.5.3q.<br>- Light-sheet microscope data was visualized using Huygens (Scientific Volume Imaging, Netherlands) version 22.10.<br>- SEAP and RT-qPCR data were calculated using Microsoft Excel 16, and averaged including calculating the error estimates using Python 3.11 with the seaborn 0.11.2 package; independent Student's t-tests were performed with Python 3.11 and the statannotations 0.6.0 and scipy 1.13.1 packages.<br>- Dose response curves were fit using Python 3.11 and the scipy 1.13.1 package.<br>- The analysis of DNA sequences and the design of plasmid maps and oligonucleotide sequences were performed with Geneious 10 (Biomatters, New Zealand) and Benchling (Benchling, USA).

For manuscripts utilizing custom algorithms or software that are central to the research but not yet described in published literature, software must be made available to editors and reviewers. We strongly encourage code deposition in a community repository (e.g. GitHub). See the Nature Portfolio guidelines for submitting code & software for further information.

Data

Policy information about availability of data

All manuscripts must include a data availability statement. This statement should provide the following information, where applicable:

- Accession codes, unique identifiers, or web links for publicly available datasets
- A description of any restrictions on data availability
- For clinical datasets or third party data, please ensure that the statement adheres to our policy

All data generated in this study are provided in the Supplementary Information, the Source Data file, and the Supplementary files contained in the archive Supplementary Data 1.zip. Source data are provided with this paper. Further raw and associated data that support the findings of this study are available from the corresponding author upon request. The sequence maps of relevant plasmids are included in the Supplementary Information (Supplementary Data 1.zip). Materials are available on request.

Research involving human participants, their data, or biological material

Policy information about studies with human participants or human data. See also policy information about sex, gender (identity/presentation), and sexual orientation and race, ethnicity and racism.

Reporting on sex and gender	*Use the terms sex (biological attribute) and gender (shaped by social and cultural circumstances) carefully in order to avoid confusing both terms. Indicate if findings apply to only one sex or gender; describe whether sex and gender were considered in study design; whether sex and/or gender was determined based on self-reporting or assigned and methods used.*<br>*Provide in the source data disaggregated sex and gender data, where this information has been collected, and if consent has been obtained for sharing of individual-level data; provide overall numbers in this Reporting Summary. Please state if this information has not been collected.*<br>*Report sex- and gender-based analyses where performed, justify reasons for lack of sex- and gender-based analysis.*
Reporting on race, ethnicity, or other socially relevant groupings	*Please specify the socially constructed or socially relevant categorization variable(s) used in your manuscript and explain why they were used. Please note that such variables should not be used as proxies for other socially constructed/relevant variables (for example, race or ethnicity should not be used as a proxy for socioeconomic status).*<br>*Provide clear definitions of the relevant terms used, how they were provided (by the participants/respondents, the researchers, or third parties), and the method(s) used to classify people into the different categories (e.g. self-report, census or administrative data, social media data, etc.)*<br>*Please provide details about how you controlled for confounding variables in your analyses.*
Population characteristics	*Describe the covariate-relevant population characteristics of the human research participants (e.g. age, genotypic information, past and current diagnosis and treatment categories). If you filled out the behavioural & social sciences study design questions and have nothing to add here, write "See above."*
Recruitment	*Describe how participants were recruited. Outline any potential self-selection bias or other biases that may be present and how these are likely to impact results.*
Ethics oversight	*Identify the organization(s) that approved the study protocol.*

Note that full information on the approval of the study protocol must also be provided in the manuscript.

Field-specific reporting

Please select the one below that is the best fit for your research. If you are not sure, read the appropriate sections before making your selection.

☒ Life sciences ☐ Behavioural & social sciences ☐ Ecological, evolutionary & environmental sciences

For a reference copy of the document with all sections, see nature.com/documents/nr-reporting-summary-flat.pdf

Life sciences study design

All studies must disclose on these points even when the disclosure is negative.

Sample size

- The sample size was determined based on our experience of optogenetic experimental setups, and the exact sizes (n) for averaged data were provided in each figure legend. (references: Nat Methods. 2020 Jul;17(7):717-725. and Nat Biotechnol. 2022 Feb;40(2):262-272.). Further specification on key experiments below:

- Optogenetic gene expression experiments using the SEAP gene were generally performed in four biological replicates if not stated otherwise in the figure legends, either representing cultures of a cell line or originating from individual plasmid transfections, where each sample was seeded at the same time and the same cell count and was transfectied with plasmid DNA individually. For the 455 nm light sample in Figure 2C with 1/10 of the SEAP reporter constructs, and the 660 nm light sample in HEK-293T cells with C,Rt in Figure S2B, Supplementary Information, three values were used (see below).

- Transcript/mRNA quantifications of optogenetic WNT3A expression (Figure 6B), for each sample or illumination condition, cell samples were taken, RNA extracted and cDNA generated. For each sample, three technical replicates were used for the transcript quantification.

- For screening purpose of the optogenetic response profiles of randomly-selected cell clones (Figure 3 B and D), a single cell sample for each illumination condition was used which was measured twice with different dilution factors (two technical replicates) and averaged.

- Microscopic analysis of optogenetic necroptosis induction with all-over illumination in Figure 4B shows one out of seven independent repeats.

- Dose-response curves using the SEAP reporter (Figure 3E) show data of 4 biological replicates for each light intensity. Dose-response curves for the induction of necroptosis (Figure 4C) integrates data from three biological culture replicates where cells were quantified in an area of 1.76 mm2 accounting to 4979   466 cells in the dark samples.

- The experiment with 3D spheroid cultures and all-over illumination for the optogenetic induction of necroptosis (Figure 4D), shows representative data of one out of three independent experiments with similar results. The experiment with 3D spheroids for the optogenetic induction of Wnt signaling (Figure 6D), shows representative samples of one out of three similar experiments, with 18 illuminated and four dark incubated individually imaged samples.

- For imaging experiments using 3D-printed photomasks for the spatial induction of optogenetic cells (Figure 5D), six samples were used and illuminated with blue light intensities ranging form 20 µmol m-2 s-1 to 70 µmol m-2 s-1 and an erythromycin control for each intensity (see Figure S5).

- Live cell imaging experiments with the optogenetic induction (DMD or light sheet) are intrinsically limited to a single specimen per experiment due to the need for constant focused illumination over the course of the experiment. However, we validated the function of the used cell lines in preceding characterization experiments and in successive optimization experiments to identify suitable experimental parameters. The number of experimental repeats (one to four times) is provided in the figure legends.S

Data exclusions

For the 455 nm light sample in Figure 2C with 1/10 of the SEAP reporter constructs, and the 660 nm light sample in HEK-293T cells with C,Rt in Figure S2B, Supplementary Information, three values were used (instead of four values as for the other samples) due to a pipetting error.

Replication

Experiments were performed multiple times in similar formats to ensure reproducibility, except for screening experiments (testing of cell clones immediately after isolation as in Figure 3B,C, Figure S9). However, the selected cell lines as the outcome of the screening were re-tested, characterized, and used in further experiments. Live imaging experiments using the DMD device with a limitation for a single sample were validated as reproducible during optimization experiments with slight modifications such as adjusting the light intensities or the projection patterns).

Randomization

No randomization was performed.

Blinding

No blinding was performed.

Reporting for specific materials, systems and methods

We require information from authors about some types of materials, experimental systems and methods used in many studies. Here, indicate whether each material, system or method listed is relevant to your study. If you are not sure if a list item applies to your research, read the appropriate section before selecting a response.

Materials & experimental systems

n/a	Involved in the study	
x		Antibodies
	x	Eukaryotic cell lines
x		Palaeontology and archaeology
x		Animals and other organisms
x		Clinical data
x		Dual use research of concern
x		Plants

Methods

n/a	Involved in the study	
x		ChIP-seq
x		Flow cytometry
x		MRI-based neuroimaging

Eukaryotic cell lines

Policy information about cell lines and Sex and Gender in Research

Cell line source(s)	Chinese hamster ovary CHO-K1 (DSMZ, ACC 110)<br>Human embryonic kidney HEK-293 (DSMZ, ACC 305)<br>Human embryonic kidney HEK-293T (DSMZ, ACC 635)<br>HEK-293T-derived TOP-GFP (Wesslowski, J. et al. J. Biol. Chem. 295, 8759–8774 (2020))<br>HEKCdh3 (Glykofrydis, F., Cachat, E., Berzanskyte, I., Dzierzak, E. & Davies, J. A. ACS Synth. Biol. 10, 1465–1480 (2021).)<br>HeLa (ATCC, strain number CCL-2)
Authentication	Cell line information available at:<br>American Type Culture Collection (ATCC), www.atcc.org<br>German Collection of Microorganisms and Cell Cultures (DSMZ), Braunschweig, Germany, www.dsmz.de<br>Cell lines were authenticated by ATCC and DSMZ, not by us.
Mycoplasma contamination	The used/generated cell lines were not routinely tested for for mycoplasma contamination.
Commonly misidentified lines<br>(See ICLAC register)	No commonly misidentified cell lines were used in this study.

Plants

Seed stocks	*Report on the source of all seed stocks or other plant material used. If applicable, state the seed stock centre and catalogue number. If plant specimens were collected from the field, describe the collection location, date and sampling procedures.*
Novel plant genotypes	*Describe the methods by which all novel plant genotypes were produced. This includes those generated by transgenic approaches, gene editing, chemical/radiation-based mutagenesis and hybridization. For transgenic lines, describe the transformation method, the number of independent lines analyzed and the generation upon which experiments were performed. For gene-edited lines, describe the editor used, the endogenous sequence targeted for editing, the targeting guide RNA sequence (if applicable) and how the editor was applied.*
Authentication	*Describe any authentication procedures for each seed stock used or novel genotype generated. Describe any experiments used to assess the effect of a mutation and, where applicable, how potential secondary effects (e.g. second site T-DNA insertions, mosiacism, off-target gene editing) were examined.*
